# Simvastatin and ROCK Inhibitor Y-27632 Inhibit Myofibroblast Differentiation of Graves’ Ophthalmopathy-Derived Orbital Fibroblasts *via* RhoA-Mediated ERK and p38 Signaling Pathways

**DOI:** 10.3389/fendo.2020.607968

**Published:** 2021-02-01

**Authors:** Yi-Hsuan Wei, Shu-Lang Liao, Sen-Hsu Wang, Chia-Chun Wang, Chang-Hao Yang

**Affiliations:** ^1^ Department of Ophthalmology, National Taiwan University Hospital, Taipei, Taiwan; ^2^ Graduate Institute of Clinical Medicine, College of Medicine, National Taiwan University, Taipei, Taiwan; ^3^ Department of Ophthalmology, College of Medicine, National Taiwan University, Taipei, Taiwan

**Keywords:** simvastatin, Y-27632, Ras homolog family member A (RhoA), Rho‑associated protein kinase (ROCK), myofibroblast, Graves’ ophthalmopathy, ERK, p38

## Abstract

Transforming growth factor-β (TGF-β)-induced differentiation of orbital fibroblasts into myofibroblasts is an important pathogenesis of Graves’ ophthalmopathy (GO) and leads to orbital tissue fibrosis. In the present study, we explored the antifibrotic effects of simvastatin and ROCK inhibitor Y-27632 in primary cultured GO orbital fibroblasts and tried to explain the molecular mechanisms behind these effects. Both simvastatin and Y-27632 inhibited TGF-β-induced α-smooth muscle actin (α-SMA) expression, which serves as a marker of fibrosis. The inhibitory effect of simvastatin on TGF-β-induced RhoA, ROCK1, and α-SMA expression could be reversed by geranylgeranyl pyrophosphate, an intermediate in the biosynthesis of cholesterol. This suggested that the mechanism of simvastatin-mediated antifibrotic effects may involve RhoA/ROCK signaling. Furthermore, simvastatin and Y-27632 suppressed TGF-β-induced phosphorylation of ERK and p38. The TGF-β-mediated α-SMA expression was suppressed by pharmacological inhibitors of p38 and ERK. These results suggested that simvastatin inhibits TGF-β-induced myofibroblast differentiation *via* suppression of the RhoA/ROCK/ERK and p38 MAPK signaling pathways. Thus, our study provides evidence that simvastatin and ROCK inhibitors may be potential therapeutic drugs for the prevention and treatment of orbital fibrosis in GO.

## Introduction

Graves’ ophthalmopathy (GO) is the ocular manifestation of Graves’ disease (GD) and occurs in 25–50% of GD patients (1). It is characterized by inflammation and fibrosis of the orbital tissue, including the retrobulbar fat, connective tissue, and extraocular muscles (2). The expansion of the orbital tissue displaces the orbital globe by pushing it forward and results in clinical signs such as proptosis, lagophthalmos, and even sight-threatening exposure keratopathy or compressive optic neuropathy (1). Fibrosis of orbital tissue and enlargement of extraocular muscles may lead to strabismus and diplopia, which strongly affect the quality of life of patients suffering from GO (3). Orbital fibroblasts appear to play a central role in the pathogenesis of GO. The thyroid-stimulating hormone receptor and the insulin-like growth factor receptor on orbital fibroblasts are the major autoantigens responsible for GO (4). Once activated, orbital fibroblasts may generate numerous inflammatory molecules and differentiate into adipocytes or myofibroblasts, leading to tissue remolding in GO (5).

Orbital fibrosis is an important pathological change that occurs in GO (6). Transforming growth factor-β (TGF-β) plays a pivotal role in the pathophysiology of many fibrotic disorders, including GO (7). In previous studies, TGF-β has been used to induce extracellular matrix production and myofibroblast differentiation in GO orbital fibroblasts. The expression of fibrotic markers, including that of α-smooth muscle actin (α-SMA), connective tissue growth factor (CTGF), fibronectin, and collagen, was increased after the stimulation of TGF-β in GO orbital fibroblasts (7).

Statins are a class of drugs that are commonly used to lower serum cholesterol and prevent the development of cardiovascular diseases by inhibiting the enzyme hydroxymethylglutaryl-coenzyme A (HMG-CoA) reductase, which plays a crucial role in cholesterol synthesis. Statins also show pleiotropic effects e.g., anti-inflammatory, antifibrotic, and immune-modulatory (8, 9). In a recent database study among GD patients, the use of statins was associated with a reduced chance of developing GO (10). However, the precise molecular mechanism for it has not been fully established. Statins exert their antifibrotic effects on several organs, such as heart, lungs, and intestines (9, 11–13). In this study, we aimed to explore the antifibrotic effect of statins in GO to explain the possible molecular mechanisms through which statins reduce/inhibit GO development.

Ras homolog family member A (RhoA) plays an essential role in cell adhesion, migration, and transformation by organizing the actin cytoskeleton (14) and the Rho-associated protein kinase (ROCK) is an important downstream effector of RhoA. There are two ROCK isoforms, ROCK1 and ROCK2, that are widely distributed in whole body tissues (15). The RhoA/ROCK signaling may play an important role in TGF-β signaling to control myofibroblast differentiation (16). Inhibition of the RhoA/ROCK pathway could reduce the TGF-β-induced expression of α-SMA, CTGF, and collagen I, as well as the myofibroblast differentiation of fibroblasts (17–19). Statins inhibit the synthesis of intermediates of cholesterol biosynthesis such as farnesyl pyrophosphate (FPP) and geranylgeranyl pyrophosphate (GGPP), which are essential for the post-translational modification of the Rho family proteins (8). Therefore, statins have a potential therapeutic role in fibrotic diseases owing to their inhibitory effects on RhoA/ROCK signaling (20, 21). Simvastatin, a type of statin, can suppress TGF-β-induced myofibroblast transformation of fibroblasts through RhoA/ROCK inhibition (20, 21).

In this study, we investigated the effects of simvastatin and ROCK inhibitor Y-27632 on TGF-β1-induced α-SMA production in cultured GO orbital fibroblasts. We also explored the possible pathways through which simvastatin and ROCK inhibitor Y-27632 affect TGF-β1 signaling. We hypothesized that simvastatin and ROCK inhibitor Y-27632 could inhibit the myofibroblast differentiation of GO orbital fibroblasts, as well as the process of fibrosis in GO.

## Materials and Methods

### Orbital Tissue Collection

Orbital tissue specimens were collected from the surgical waste produced during the orbital decompression surgeries of six GO patients. The clinical characteristics of the patients are listed in **Table 1**. All patients were euthyroid, with a clinical activity score of less than three for more than 6 months prior to the surgery. None of them had received radiotherapy or steroid pulse therapy in the past. Patient recruitment for the study was carried out at the Department of Ophthalmology, National Taiwan University Hospital, Taiwan. The protocol was approved by the Institutional Review Board of National Taiwan University Hospital, Taiwan (201908011RINA). Informed consent was obtained from all participants in accordance with the Declaration of Helsinki and Good Clinical Practice.

**Table 1:** Clinical characteristics of the patients included in this study.

Age, years	Sex	Duration of GO, months	CAS	fT4 (ng/dL)	TSH (mU/L)	TBII (%)	Previous treatment for GO
34	F	12	2	1.36	0.48	61.2	None
38	M	10	1	1.14	1.17	19.7	None
41	F	28	1	1.01	1.96	24.3	None
47	F	22	2	0.82	2.07	38.3	Oral steroids
57	F	20	1	1.09	1.39	54.2	None
58	M	36	2	0.91	0.57	44.5	Oral steroids

CAS, clinical activity score; fT4, free thyroxine; TSH, thyroid stimulating hormone; TBII, thyrotropin binding inhibitory immunoglobulin.

### Orbital Fibroblast Culture and Stimulation

Orbital fibroblasts were cultivated as previously reported (22). Orbital tissues were minced and placed in plastic culture dishes with Dulbecco’s modified Eagle’s medium (DMEM) supplemented with 20% fetal bovine serum (FBS) and 50 IU/mL penicillin-streptomycin (Gibco^®^; Thermo Fisher Scientific, Waltham, MA, USA), allowing orbital fibroblasts to grow out. Monolayers were covered with DMEM supplemented with 10% FBS and serially passaged with gentle trypsin/ethylenediaminetetraacetic acid (EDTA) (Gibco^®^; Thermo Fisher Scientific, Waltham, MA, USA) treatment. These were incubated in a humidified incubator at 37°C and 5% carbon dioxide (CO_2_).

The orbital fibroblasts were seeded into six-well plates at a density of 2 × 10^5^ cells per well and stimulated at 90% confluence with 3 ng/mL of recombinant human TGF-β1 (Cat. No. 580701; BioLegend, San Diego, CA, USA) with or without pretreatment with simvastatin (TargetMol, Boston, MA, USA) or ROCK inhibitor Y-27632 (Sigma-Aldrich, St. Louis, MO, USA). In some experiments, fibroblasts were pretreated with PD98059 (ERK inhibitor) and SB203580 (p38 inhibitor) obtained from Cell Signaling Technology (Beverly, MA, USA) before stimulation with TGF-β1. We purchased FPP ammonium salt (F6892), GGPP ammonium salt (G6025), and mevalonate (90469) from Sigma (St. Louis, MO, USA) for cell stimulation in some experiments. GGTI-298 (Cat. No. 2430) and FTI-277 (Cat. No. 2407) were purchased from Tocris Bioscience (Bristol, United Kingdom). All experiments were performed with fibroblasts between the third and eighth passages from culture initiation. Six independent strains from different donors were used for the repeated experiments.

### Immunofluorescence Staining

The orbital fibroblasts were cultured on six-well plates containing glass coverslips, and the cells were fixed and blocked for the immunofluorescence assay. The coverslips were incubated with anti-α-SMA primary antibody ab5694 (Abcam, Cambridge, MA, USA) overnight at 4°C. After three washes, the coverslips were processed with VectaFluor Excel Amplified Anti-Rabbit IgG Kit, DyLight^®^ 488 Antibody Kit (DK-1488; Vector Laboratories, Burlingame, CA, USA). The nuclei were counterstained using H-1500 DAPI (Vector Laboratories, Burlingame, CA, USA). The glass slides were then observed with a fluorescence microscope coupled to a CCD camera (EVOS FLc; Life Technologies, Carlsbad, CA, USA). The fluorescence intensity quantification was performed using ImageJ.

### Real-Time Polymerase Chain Reaction

Total RNA was extracted from cultured orbital fibroblasts using the TRIzol reagent (Invitrogen; Carlsbad, CA, USA). Complementary DNA (cDNA) was synthesized using 1 μg of RNA according to the manufacturer’s instructions for the iScript cDNA Synthesis Kit (Bio-Rad, Hercules, CA, USA). Real-time PCR was performed on a thermocycler (StepOne Real-Time PCR System; Applied Biosystems, Foster City, CA, USA) using SYBR Green Master Mix (Applied Biosystems, Foster City, CA, USA). All PCRs were performed in triplicate. The sequences of the primers used were as follows: α-SMA forward: 5′-CTC CCA GGG CTG TTT TCC CA-3′, reverse: 5′-CCA TGT CGT CCC AGT TGG TG-3′; CTGF forward: 5′-TGT GTG ACG AGC CCA AGG A-3′, reverse: 5′-TCT GGG CCA AAC GTG TCT TC-3’; and Fibronectin 1 (FN1) forward: 5’-CCA AGA AGG GCT CGT GTG A-3′, reverse: 5′-TGG CTG GAA CGG CAT CA-3′. The primers for glyceraldehyde 3-phosphate dehydrogenase (GAPDH) were obtained from Sino Biological, Inc. (Cat no. HP100003; Wayne, PA, USA). The messenger RNA (mRNA) levels of each target gene were normalized to GAPDH and represented as fold changes.

### Western Blot Analysis

Proteins were extracted from tissue homogenates and cell lysates. After collecting the protein samples, equal amounts of protein (50 μg) were boiled in a sample buffer. The protein samples were separated in a 10% sodium dodecyl sulfate-polyacrylamide gel and transferred to a nitrocellulose membrane. The blots were blocked with 4% bovine serum albumin for 1 h at room temperature and then probed with primary antibodies at 4°C overnight. The ab5694 antibody for α-SMA (1:1000 dilution) was purchased from Abcam (Cambridge, MA, USA). The antibodies for RhoA (1:1000 dilution, no. 2117), ROCK1 (1:1000 dilution, no. 4035), Smad2/3 (1:1000 dilution, no. 5678), phospho-Smad2 (Ser465/467)/Smad3 (Ser423/425) (1:1000 dilution, no. 8828), ERK1/2 (1:1000 dilution, no. 9102), phospho-ERK1/2 (Thr202/Tyr204) (1:2000 dilution, no. 4370), p38 MAPK (1:1000 dilution, no. 8212), phospho-p38 MAPK (Thr180/Tyr182) (1:1000 dilution, no. 9211), JNK (1:1000 dilution, no. 9252), and phospho-JNK (Thr183/Tyr185) (1:1000 dilution, no. 4668) were purchased from Cell Signaling Technology (Beverly, MA, USA). Immunoreactive bands were detected with horseradish peroxidase-conjugated secondary antibodies and developed using enhanced chemiluminescence detection (MilliporeSigma, Burlington, MA, USA) and exposure to X-ray film. The relative amount of each immunoreactive band was quantified using the ImageJ software and normalized to the levels of the reference molecules.

### Statistical Analyses

At least three cell strains from different individuals were used in all experiments, and all sample assays were carried out in triplicate. The experimental results are shown as the mean ± SD calculated from normalized measurements. Analysis of variance or the Student’s t-test were used to determine statistical significance (p < 0.05) using GraphPad Prism 8.4.2 (GraphPad Software, San Diego, CA, USA).

## Results

### Simvastatin and ROCK Inhibitor Y-27632 Inhibited TGF-β-Induced α-SMA Expression in GO Orbital Fibroblasts

**Figure 1:**
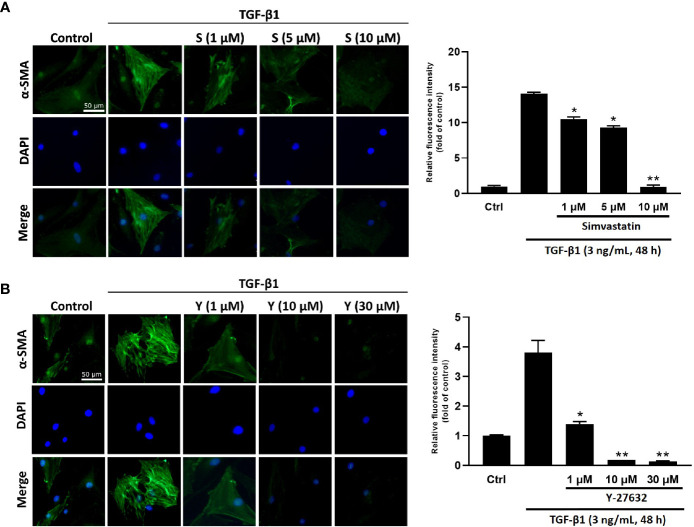
Immunocytochemical characterization of transforming growth factor-β (TGF-β)-induced α-smooth muscle actin (α-SMA) expression in Graves’ ophthalmopathy (GO) orbital fibroblasts. Primary cultured GO orbital fibroblasts were stimulated with 3 ng/mL TGF-β1 for 48 h with or without a 1-h pretreatment with different concentrations of simvastatin (1, 5, 10 μM) **(A)** or ROCK inhibitor Y-27632 (1, 10, 30 μM) **(B)**. α-SMA expression was detected using immunofluorescence staining (green). The bar charts show mean data of relative fluorescence intensity of α-SMA presented as the fold of control. *p < 0.05, **p < 0.01 compared to cells treated with TGF-β1 alone. S = simvastatin; Y = Y-27632.

To determine the effect of simvastatin and ROCK inhibitor Y-27632 on TGF-β-induced myofibroblast differentiation, immunofluorescence staining for α-SMA (marker for myofibroblast) was performed in cultured GO orbital fibroblasts. The expression of α-SMA and actin filament formation were markedly increased after TGF-β1 (3 ng/mL) stimulation for 48 h (**Figure 1**). However, pretreatment of the cells with different concentrations of simvastatin (1, 5, 10 μM) (**Figure 1A**) or ROCK inhibitor Y-27632 (1, 10, 30 μM) (**Figure 1B**) diminished TGF-β-induced α-SMA expression in orbital fibroblasts.

**Figure 2:**
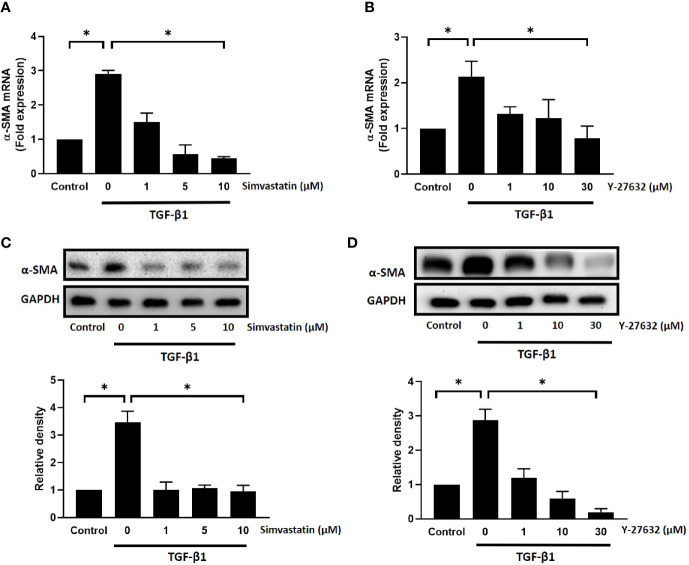
Effect of simvastatin and ROCK inhibitor Y-27632 on transforming growth factor-β (TGF-β)-induced α-smooth muscle actin (α-SMA) messenger RNA (mRNA) and protein expression in Graves’ ophthalmopathy (GO) orbital fibroblasts. Primary cultured GO orbital fibroblasts were stimulated with TGF-β1 (3 ng/mL) for 48 h with or without a 1-h pretreatment with different concentrations of simvastatin (1, 5, 10 μM) **(A, C)** or ROCK inhibitor Y-27632 (1, 10, 30 μM) **(B, D)**. The α-SMA mRNA expression was examined using real-time PCR **(A, B)**. The α-SMA protein production was determined using western blot analysis **(C, D)**. Data are presented as mean ± SD from at least three independent experiments. *p < 0.05.

To further confirm the results, we used real-time PCR and western blot analysis to assess the mRNA and protein expression levels of α-SMA, respectively. As shown in **Figure 2**, TGF-β1 significantly induced an increase in mRNA and protein expression levels of α-SMA in GO orbital fibroblasts. Pretreatment with different concentrations of simvastatin (1, 5, 10 μM) (**Figures 2A, C**) or ROCK inhibitor Y-27632 (1, 10, 30 μM) (**Figures 2B, D**) significantly inhibited TGF-β-induced α-SMA mRNA expression and protein production. The inhibitory effect of simvastatin and Y-27632 appeared to be dose-dependent. Therefore, 10 and 30 μM concentrations of simvastatin and Y-27632, respectively, were used in the subsequent experiments to achieve a more prominent effect. As α-SMA is the most common marker for fibroblast-to-myofibroblast differentiation, these results suggested that simvastatin and ROCK-inhibitor Y-27632 have an inhibitory effect on the TGF-β-induced differentiation of orbital fibroblasts into myofibroblasts.

### Inhibitory Effect of Simvastatin on TGF-β-Induced RhoA, ROCK1, and α-SMA Expression Was Reversed by GGPP

**Figure 3:**
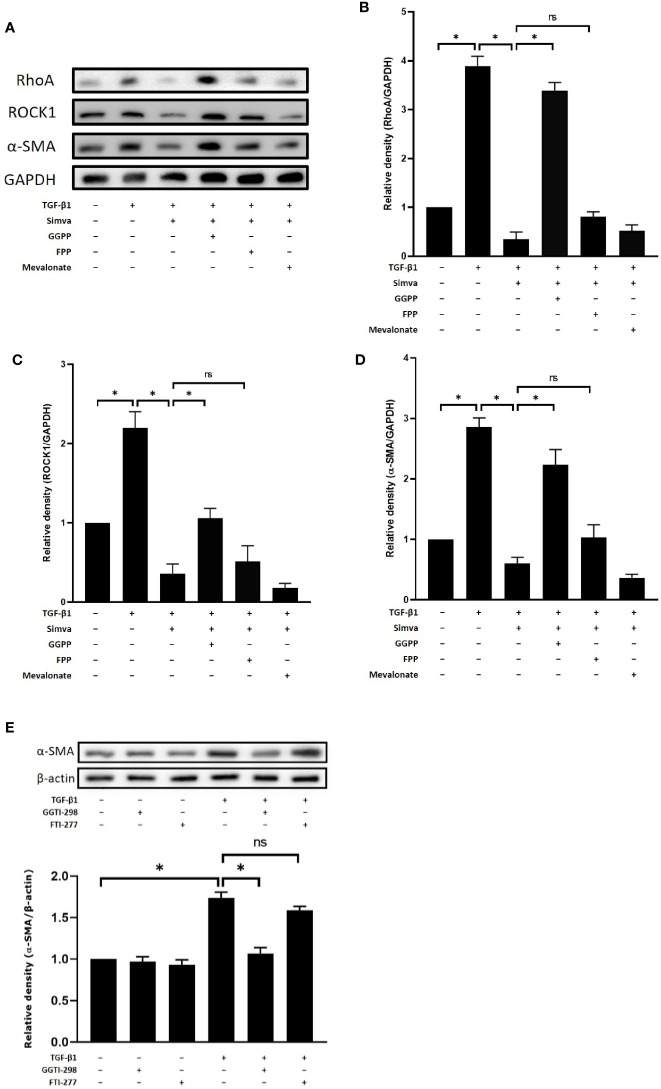
Effects of hydroxymethylglutaryl-coenzyme A (HMG-CoA) downstream intermediates on simvastatin-mediated inhibition of transforming growth factor-β (TGF-β)-induced RhoA, Rho-associated protein kinase 1 (ROCK1), and α-smooth muscle actin (α-SMA) expression. **(A)** Graves’ ophthalmopathy (GO) orbital fibroblasts were stimulated with TGF-β1 (3 ng/mL) for 48 h with or without a 1-h pretreatment with simvastatin (10 μM) and addition of geranylgeranyl pyrophosphate (GGPP) (10 μM), farnesyl pyrophosphate (FPP) (10 μM), or mevalonate (200 μM). The protein levels of RhoA, ROCK1, and α-SMA were determined using western blot analysis. **(B–D)** The densities of RhoA **(B)**, ROCK1 **(C)**, and α-SMA **(D)** protein bands were quantified and normalized to GAPDH. **(E)** GO orbital fibroblasts were stimulated with TGF-β1 (3 ng/mL) for 48 h with or without a 1-h pretreatment with geranylgeranyl transferase inhibitor (GGTI-298) (10 μM) or farnesyl transferase inhibitor (FTI-227) (10 μM). The α-SMA protein production was determined using western blot analysis. Data are presented as mean ± SD of at least three independent experiments. *p < 0.05.

Simvastatin, an HMG-CoA reductase, inhibits the synthesis of mevalonate and other precursors of cholesterol such as FPP and GGPP, which are important for farnesylation and geranylgeranylation of Rho proteins. To investigate the mechanism of simvastatin-mediated inhibition of myofibroblast differentiation, we studied the effects of the HMG-CoA downstream intermediates, mevalonate, FPP, and GGPP, on the expressions of RhoA, ROCK1, and α-SMA. As shown in **Figures 3A–D**, simvastatin inhibited TGF-β1-induced expression of RhoA, ROCK1, and α-SMA in GO orbital fibroblasts. The addition of 10 μM of GGPP reversed the inhibitory effects of simvastatin on RhoA, ROCK1, and α-SMA expression. However, the addition of FPP or mevalonate failed to prevent the suppressive effects of simvastatin. Moreover, the geranylgeranyl transferase inhibitor (GGTI-298), not the farnesyl transferase inhibitor (FTI-227), showed simvastatin-like inhibition of TGF-β1-induced α-SMA (**Figure 3E**). Based on these results, we propose that the mechanism of simvastatin-mediated inhibition of myofibroblast differentiation may involve RhoA/ROCK signaling. Simvastatin may inhibit the TGF-β1-induced RhoA/ROCK signaling by blocking Rho geranylgeranylation, but not Rho farnesylation.

### Simvastatin and ROCK Inhibitor Y-27632 Inhibited TGF-β-Induced Phosphorylation of ERK and p38, But Not That of Smad

**Figure 4:**
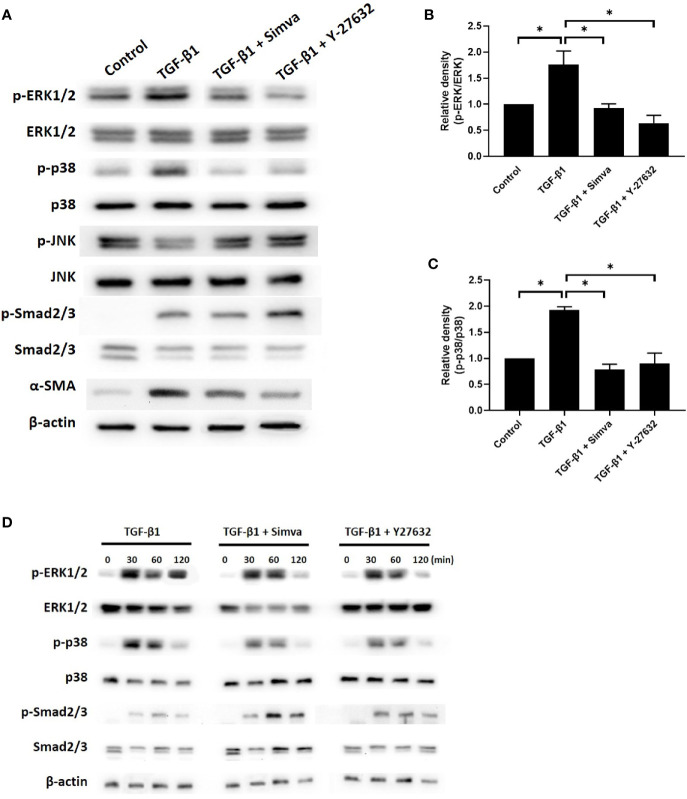
Effects of simvastatin and ROCK inhibitor Y-27632 on transforming growth factor-β (TGF-β)-induced signaling. **(A)** Primary cultured Graves’ ophthalmopathy (GO) orbital fibroblasts were stimulated with TGF-β1 (3 ng/mL) for 48 h with or without a 1-h pretreatment with simvastatin (10 μM) or ROCK inhibitor Y-27632 (30 μM). The expression and phosphorylation levels of Smad2/3, ERK1/2, p38, and JNK were determined using western blot analysis. **(B, C)** The densities of ERK1/2 **(B)** and p38 **(C)** protein bands were quantified and normalized to β-actin. **(D)** GO orbital fibroblasts were stimulated with TGF-β1 (3 ng/mL) for 30, 60, 120 min with or without a 1-h pretreatment with simvastatin (10 μM) or ROCK inhibitor Y-27632 (30 μM). The expression and phosphorylation levels of Smad2/3, ERK1/2, and p38 were determined using western blot analysis. Data are presented as mean ± SD of at least three independent experiments. *p < 0.05.

The signaling pathways implicated in TGF-β-induced myofibroblast differentiation include the Smad and non-Smad pathways, which incorporate the different branches of MAPK signaling (16). To explore the effects of simvastatin and ROCK inhibitor Y-27632 on TGF-β signaling in GO orbital fibroblasts, we investigated whether simvastatin and Y-27632 could inhibit TGF-β-induced phosphorylation of Smad2/3, ERK1/2, p38, and JNK. As shown in **Figures 4A–C**, 48 h of 3 ng/mL TGF-β treatment induced phosphorylation of Smad2/3, ERK1/2, and p38 in GO orbital fibroblasts. Simvastatin and Y-27632 successfully abrogated TGF-β-induced phosphorylation of ERK1/2 and p38. In contrast, they had no significant impact on TGF-β-induced phosphorylation of Smad2/3. Since the Smad and MAPK pathway could usually be activated in several minutes, we also assessed their phosphorylated states at early time-points (30, 60, 120 min) after TGF-β stimulation (**Figure 4D**). The results consistently demonstrated that simvastatin and Y-27632 inhibited TGF-β-induced phosphorylation of ERK1/2 and p38, but not Smad2/3. Our data suggested that simvastatin and Y-27632 could both inhibit the early- and late-phase activation of TGF-β-induced ERK and p38 signaling, but not Smad pathway in GO orbital fibroblasts.

### Simvastatin and ROCK Inhibitor Y-27632 Inhibited TGF-β-Induced α-SMA Expression by Blocking ERK and p38 Signaling

**Figure 5:**
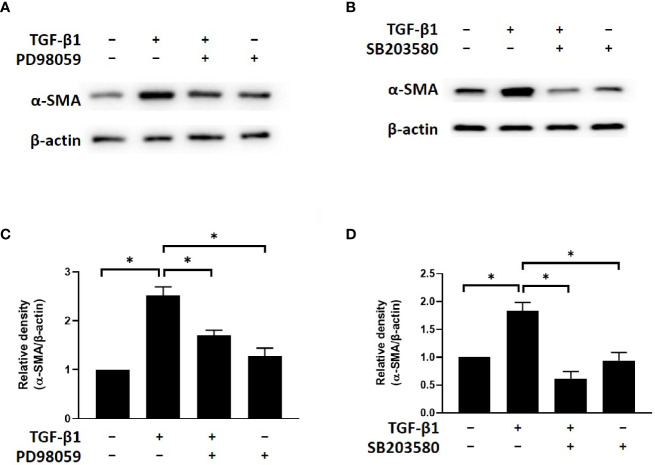
Effects of ERK and p38 inhibitors on TGF-β-induced α-SMA expression in Graves’s ophthalmopathy (GO) orbital fibroblasts. Primary cultured GO orbital fibroblasts were stimulated with transforming growth factor-β (TGF-β1) (3 ng/mL) for 48 h with or without a 1-h pretreatment with 10 μM of PD98059 (ERK inhibitor) **(A, C)** and 10 μM of SB203580 (p38 inhibitor) **(B, D)**. The protein levels of α-SMA were determined using western blot analysis. The densities of α-SMA protein bands were quantified and normalized to β-actin. Data are presented as mean ± SD of at least three independent experiments. *p < 0.05.

To determine the potential involvement of ERK and p38 signaling in the mechanism by which simvastatin and Y-27632 inhibit myofibroblast differentiation, we investigated the effects of PD98059 (ERK inhibitor) and SB203580 (p38 inhibitor) on TGF-β-induced α-SMA expression. The results of western blot analysis showed that TGF-β-induced α-SMA expression in GO orbital fibroblasts was suppressed by PD98059 (**Figures 5A, C**) and SB203580 (**Figures 5B, D**). Based on previous data, simvastatin and Y-27632 have similar effects of ERK inhibitor and p38 inhibitor to block TGF-β-induced ERK and p38 signaling. These results suggested that simvastatin and Y-27632 inhibited TGF-β-induced α-SMA expression by blocking ERK and p38 signaling as well.

## Discussion

Orbital connective tissue remodeling and fibrosis are characteristic processes that appear relatively late in the clinical course of GO. The TGF-β produced by GO orbital fibroblasts may stimulate excessive production of extracellular matrix and differentiation of Thy-1-positive orbital fibroblasts into myofibroblasts, which express strong α-SMA immunoreactivity (22, 23). In this study, we showed that simvastatin and ROCK inhibitor Y-27632 inhibited the expression of TGF-β-induced α-SMA, which serves as a marker for fibrosis and indicates myofibroblast differentiation.

Some studies have demonstrated the inhibitory effects of simvastatin and Y-27632 on TGF-β-induced myofibroblast differentiation from fibroblasts derived from different disease specimens, such as nasal polyps (24), keloids (20), and penile tunica albuginea from Peyronie’s disease (21). To the best of our knowledge, this is the first study to investigate the antifibrotic effects of simvastatin and Y-27632 in GO-derived fibroblasts. Further investigations on any synergistic effects of these two components will be helpful to understand their antifibrotic properties and potential therapeutic roles in GO.

Statins are commonly used to prevent coronary artery disease and stroke by reducing low-density lipoprotein cholesterol levels. Recently, the pleiotropic antifibrotic effects of statins have been proposed in various organ systems, such as heart (9, 11), liver (25, 26), and lungs (27, 28). Also, the possible protective role of statins in GO has been proposed (29). Statin usage is believed to be associated with a reduced risk of developing GO among patients with GD in a large cohort study (10). They found that patients who used statins for at least 60 d during the period of observation had a 40% lower risk of developing GO. The precise molecular mechanisms by which statins reduce GO risk are not fully established. Some evidence suggests that statins may modulate both apoptosis and autophagic activities in patients with GD (29). This elucidation was based on the involvement of cellular apoptosis and autophagy in the pathogenesis of GO (30, 31). In addition, a recent study revealed that simvastatin may inhibit adipogenesis, as well as the expression of early and late adipogenic genes in human orbital fibroblasts (32). Our results provided *in vitro* evidence of simvastatin-mediated antifibrotic effects in GO that may also explain the possible protective effect of simvastatin against the development of GO.

The possible mechanism underlying statin-mediated antifibrotic effects is the inhibition of geranylgeranylated Rho protein, which in turn inhibits the Rho/ROCK signaling pathway (33, 34). Statins inhibit HMG-CoA reductase, the catalyst for the synthesis of mevalonate from HMG-CoA. This inhibition leads to a reduction of downstream intermediate compounds, including the isoprenoid GGPP and FPP. These molecules are necessary for the posttranslational modification of the Rho proteins, which is crucial for the Rho proteins to play their proper functions. In human keloid fibroblasts, simvastatin inhibited TGF-β-induced RhoA activation and RhoA/ROCK signaling by interfering with posttranslational geranylgeranylation of RhoA (20). In human airway fibroblasts, the inhibitory effects of simvastatin on TGF-β-induced fibronectin could be reversed by the addition of either GGPP or FPP (28). The study of human tenon fibroblasts suggested that the inhibition of Rho-geranylgeranylation, not Rho-farnesylation, was the mechanism for lovastatin to inhibit myofibroblast differentiation (35). In our study, we found that only GGPP, and not FPP, could reverse the simvastatin-mediated inhibition of TGF-β-induced α-SMA.

Consistently, only the geranylgeranyl transferase inhibitor (GGTI-298), not the farnesyl transferase inhibitor (FTI-227), showed simvastatin-like inhibition of TGF-β1-induced α-SMA. These findings suggested that geranylgeranylation rather than farnesylation of RhoA is crucial for TGF-β-induced α-SMA expression in GO orbital fibroblasts. And simvastatin may inhibit the TGF-β1-induced RhoA/ROCK signaling by blocking Rho geranylgeranylation, but not Rho farnesylation.

The RhoA/ROCK signaling pathway is known to regulate numerous cellular functions, including cell proliferation, migration, contraction, and adhesion (36). The profound involvement of the RhoA/ROCK pathway in various disease processes has made ROCK a potential therapeutic target in many kinds of diseases, e.g., cardiovascular (37), neoplastic (38), and neurologic (39). Moreover, ROCK inhibitors have been used as potential therapeutic drugs for several ophthalmic diseases, including glaucoma, corneal endothelial diseases, age-related macular degeneration, and diabetic retinopathy (40, 41). However, there are few studies that have investigated the role of Rho/ROCK signaling or that of ROCK inhibitors in GO. Using an *in vitro* model of GO, our study demonstrated the involvement of RhoA/ROCK signaling in TGF-β-induced myofibroblast differentiation and the antifibrotic effects of the ROCK inhibitor Y-27632. In many studies Y-27632 is a commonly used ROCK inhibitor that inhibits both ROCK1 and ROCK2 (42). Further investigations are necessary to explore the possible applications of ROCK inhibitors in the treatment of GO.

**Figure 6:**
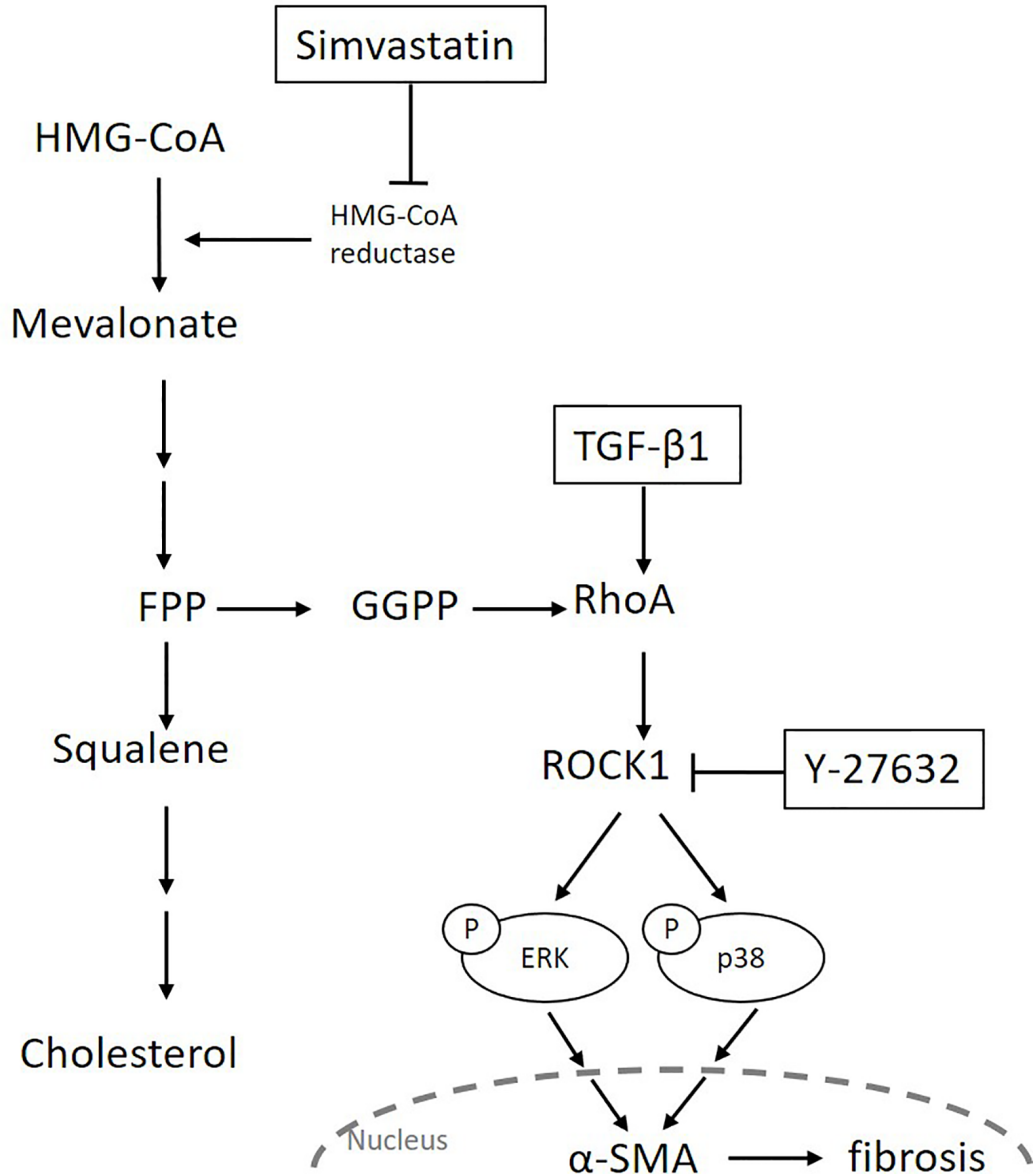
Schematic diagram of the possible signaling mechanisms underlying simvastatin- and Y-27632-mediated inhibition of transforming growth factor-β (TGF-β1)-induced orbital tissue fibrosis in Graves’ ophthalmopathy.

Transforming growth factor-β is the most potent inducer of myofibroblast differentiation and acts by activating the canonical Smad pathway or the non-Smad pathways, including Rho/ROCK signaling and different branches of the MAPK pathway (16, 43). The ROCK inhibitors were reported to inhibit TGF-β-induced myofibroblast differentiation by regulating the Smad or MAPK signaling pathways (19, 44). For example, Y-27632 suppressed TGF-β-induced phosphorylation of Smad3, but not that of Smad2, in ocular Tenon’s capsule fibroblasts (44). It also inhibited TGF-β-induced phosphorylation of ERK and JNK, but not that of p38, in renal mesangial cells (19). In our study, Y-27632 abrogated TGF-β-induced phosphorylation of ERK and p38, but not that of JNK or Smad2/3. These results indicate that ROCK mediates MAPK signaling, but each MAPK signaling is regulated distinctively in different cells or by different stimuli. In our study, simvastatin showed an effect similar to that of Y-27632 in TGF-β-induced myofibroblast differentiation. These results suggest that, although simvastatin does not regulate the TGF-β-mediated Smad pathway in GO orbital fibroblasts, it regulates the TGF-β-mediated ERK/p38 MAPK pathways, probably in a ROCK-dependent manner. In summary, we propose that simvastatin can inhibit TGF-β-induced myofibroblast differentiation *via* suppression of the RhoA/ROCK/ERK and p38 MAPK signaling pathways (**Figure 6**).

Contrary to our findings, previous studies showed that simvastatin could inhibit TGF-β-mediated Smad phosphorylation in human ventricular (11) and intestinal fibroblasts (45). We believe that the cellular mechanisms of simvastatin’s antifibrotic effect are diverse and complex in different cell types. Our data could not elucidate whether RhoA/ROCK/ERK and p38 MAPK signaling has any interaction with Smad2/3 signaling. Recently, Fang *et al*. reported that interleukin-17A can activate the JNK signaling in CD90^+^ orbital fibroblasts to promote GO fibrosis initiated by TGF-β-mediated Smad transcription (46). Further investigation is needed to understand the possible interactions between the TGF-β-induced RhoA, MAPK, and Smad pathways in GO orbital fibroblasts.

Our study also had some limitations. First, the GO orbital fibroblasts were obtained from patients with inactive GO because of the surgical indication. Further studies must be conducted using specimens from patients with active GO to compare the results between different disease stages. Second, we only managed to obtain orbital tissues from a small cohort of patients. We may need a larger sample size to validate our results in the future.

In conclusion, our results provide preliminary evidence that simvastatin and ROCK inhibitor Y-27632 inhibit TGF-β-induced differentiation of GO-derived fibroblasts into myofibroblasts. We propose that simvastatin and ROCK inhibitors may be potential candidates for the prevention and treatment of orbital tissue fibrosis in GO.

## Data Availability Statement

The raw data supporting the conclusions of this article will be made available by the authors, without undue reservation.

## Ethics Statement

The studies involving human participants were reviewed and approved by the Institutional Review Board of National Taiwan University Hospital. The patients/participants provided their written informed consent to participate in this study. No animal studies are presented in this manuscript. No potentially identifiable human images or data is presented in this study.

## Author Contributions

Y-HW, S-LL and C-CW conceptualized the experiments, designed the study, and secured funding. S-LL provided the surgical specimens. S-HW and C-CW performed the experiments and helped analyze the data. Y-HW interpreted the data and wrote the manuscript. C-HY supervised the research activity and revised the manuscript. All authors contributed to the article and approved the submitted version.

## Funding

This study was supported through research grants from National Taiwan University Hospital, Taiwan (NTUH.109-M4642).

## Conflict of Interest

The authors declare that the research was conducted in the absence of any commercial or financial relationships that could be construed as a potential conflict of interest.
